# Optical Detection of Fe^3+^ Ions in Aqueous Solution with High Selectivity and Sensitivity by Using Sulfasalazine Functionalized Microgels

**DOI:** 10.3390/s19194223

**Published:** 2019-09-28

**Authors:** Weiming Ji, Zumei Zhu, Shunni Dong, Jingjing Nie, Binyang Du

**Affiliations:** 1 MOE Key Laboratory of Macromolecular Synthesis and Functionalization, Department of Polymer Science & Engineering, Zhejiang University, Hangzhou 310027, China; jiweiming@zju.edu.cn (W.J.); 21829020@zju.edu.cn (S.D.); 2 Department of Chemistry, Zhejiang University, Hangzhou 310027, China; 21737084@zju.edu.cn (Z.Z.); niejj@zju.edu.cn (J.N.)

**Keywords:** functional microgels, sulfasalazine, Fe^3+^ detection, optical response

## Abstract

A highly selective and sensitive optical sensor was developed to colorimetric detect trace Fe^3+^ ions in aqueous solution. The sensor was the sulfasalazine (SSZ) functionalized microgels (SSZ-MGs), which were fabricated via in-situ quaternization reaction. The obtained SSZ-MGs had hydrodynamic radius of about 259 ± 24 nm with uniform size distribution at 25 °C. The SSZ-MG aqueous suspensions can selectively and sensitively response to Fe^3+^ ions in aqueous solution at 25 °C and pH of 5.6, which can be quantified by UV-visible spectroscopy and also easily distinguished by the naked eye. Job’s plot indicated that the molar binding ratio of SSZ moiety in SSZ-MGs to Fe^3+^ was close to 1:1 with an apparent association constant of 1.72 × 10^4^ M^−1^. A linear range of 0–12 μM with the detection limit of 0.110 μM (0.006 mg/L) was found. The obtained detection limit was much lower than the maximum allowance level of Fe^3+^ ions in drinking water (0.3 mg/L) regulated by the Environmental Protection Agency (EPA) of the United States. The existence of 19 other species of metal ions, namely, Ag^+^, Li^+^, Na^+^, K^+^, Ca^2+^, Ba^2+^, Cu^2+^, Ni^2+^, Mn^2+^, Pb^2+^, Zn^2+^, Cd^2+^, Co^2+^, Cr^3+^, Yb^3+^, La^3+^, Gd^3+^, Ce^3+^, and Bi^3+^, did not interfere with the detection of Fe^3+^ ions.

## 1. Introduction

With the increase of industrial activity and development of the economy, the pollution of heavy metal ions in aqueous solution has become a serious threat to ecosystems and human health. The detection of heavy metal ions and treatment of corresponding pollution are significant and challenging tasks for improving and protecting our living environments, life, and health. Regarding iron (III) ion (Fe^3+^), it is one of the heavy metal ions and at the same time also one of the important transition metals in biological systems, which is needed for many biological processes like oxygen metabolism and electron transport [1,2,3]. However, excessive levels of Fe^3+^ ions can cause some severe diseases for human body, such as Alzheimer’s [4] and Parkinson’s diseases [5]. The maximum allowance level of Fe^3+^ ions in drinking water by the Environmental Protection Agency (EPA) of the United States is 0.3 mg/L (5.4 μM). Therefore, it is important to regulate the concentration of Fe^3+^ ions in aqueous environment. Efficient chemical sensors are highly desired for the detection of Fe^3+^ ions in aqueous solutions. Efforts are devoted to develop chemical sensors with the use of fluorophores and metal nanoparticles [6] like rhodamine-based chemical sensors [7], fluorescent chemosensors [8], quantum dots [9], and so on. For example, Wei et al. [10] reported a PEGylated amphiphilic polymer (C_18_PMH-mPEG) modified core-shell upconversion nanoparticles (mPEG-UCNPs) for highly sensitive and selective detection of Fe^3+^ ions in aqueous solution with the detection limit of 89.6 nM. Kagit et al. [11] synthesized a fluorescent rhodamine-based hexapodal Fe^3+^ probe (**L**) on a cyclotriphosphazene core by an azide-alkyne “click-reaction”. The probe **L** showed remarkable fluorescence intensity enhancement when adding Fe^3+^ ions into THF/water solution of **L,** and the detection limit of **L** reached 4.8 μM (0.27 ppm) for Fe^3+^ ions. Lee et al. [12] developed a rhodamine-based Fe^3+^ detector (**FS1**) via connecting ethylene diamine and a 2-hydroxy-5-nitrobenzaldehyde. With the addition of Fe^3+^ ions, the fluorescence of **FS1** was “turn-on”, which exhibited high selectivity against the influence of other heavy metal ions with a detection limit of 0.1 μM for Fe^3+^ ions. However, most of the above-mentioned chemical sensors required complicated and time consuming synthetic techniques, which might be not available for routine analysis [6,7,8,9,10,11,12]. Therefore, it is needed and worthy to further develop simple, low-cost, efficient, yet robust chemical sensors for the detection of Fe^3+^ ions in aqueous solutions.

Microgels are porous colloidal particles with three-dimensional crosslinked network structures. The size of microgels is in the range of 10 to 1000 nm. The microgels can swollen and well disperse in aqueous solution. With proper design and the use of suitable monomers, the obtained microgels can respond chemically or physically to the changes of external environment like temperature, pH, and ionic strength, etc. [13,14,15,16,17,18,19,20]. The hydrophilic nature and porous network structures render the microgels excellent candidates for sensing applications in aqueous environment. By introducing specific functionality into the crosslinked networks, the obtained functional microgels could be applied as chemical or bio-sensors for the detection of heavy metal ions like Cu^2+^ and Pb^2+^ [21,22,23], organophosphates [24] and biomolecules like DNA, etc. [25,26,27]. 

In the present work, we report a novel sulfasalazine (SSZ) functionalized microgel for selective and sensitive detection of Fe^3+^ ions in aqueous solution. SSZ is a derivative of mesalazine (5-aminosalicylic acid) and has been used as an effective anti-inflammatory drug for the treatment of inflammatory bowel disease and rheumatoid arthritis (RA) [28] because of its safety profile, ease of administration, and low cost. The chemical structure of SSZ molecule contains carboxyl and phenolic groups as well as sulfonamide group, which are considered to be good coordinating groups with heavy metal ions. However, SSZ is hydrophobic in nature and exhibits a low solubility in water according to the Biopharmaceutics Classification Scheme (BCS), which strongly limits the possible application of SSZ in aqueous conditions [29]. Recently, we have developed a simultaneous quaternized crosslinking method during surfactant free emulsion copolymerization (SFEP) of monomers to form microgels and in-situ incorporate hydrophobic functional molecules into the crosslinked network of resultant microgels via quaternization reaction [22,23,30,31]. Herein, we successfully incorporated SSZ molecule into the microgel network via in-situ quaternization reaction with pyridine group of SSZ, leading to the SSZ functionalized microgels, named as SSZ-MGs. Transmission electron microscopy (TEM) and dynamic light scattering (DLS) were used to characterize the morphology, hydrodynamic size, and size distribution of the obtained SSZ-MGs. UV-vis absorption spectrometry was used to investigate the sensing capability of SSZ-MG aqueous suspensions toward Fe^3+^ ions in aqueous solution over the interference of other metal ions.

## 2. Materials and Methods

### 2.1. Materials 

1,6-dibromhexane (Br-C_6_H_12_-Br, 97%) was obtained from Tokyo Chemical Industry Co. Ltd., Tokyo, Japan. 2,2′-azobis(2-methylpropionamidine) dihydrochloride (AIBA, 97%) was purchased from Sigma-Aldrich. *N*-isopropylacrylamide (NIPAm, 99%), sulfasalazine (SSZ, 95%), and 1-vinylimidazole (VIM, 99%) were obtained from J&K Chemical Ltd., Shanghai, China Tris (hydroxymethyl) aminomethane (Tris-HCl, 99.8%) was purchased from Acros Organics. Sodium dihydngen phoshate anhydrous (99%) and sodium phosphate dibasic (99%) were purchased from Shanghai Macklin Biochemical Co., Ltd., Shanghai, China. Nitrate salts with twenty different metal ions were tested in the present work, whose detailed description was given in supporting information. They were obtained from J&K Chemical Ltd., Shanghai Aladdin Bio-Chem Technology Co., Ltd., and Sinopharm Chemical Reagent Co., Ltd., Shanghai, China, respectively. All of the reagents were used as received without further purification. 

### 2.2. Synthesis of Sulfasalazine Functionalized Microgels (SSZ-MGs) 

**Scheme 1:**
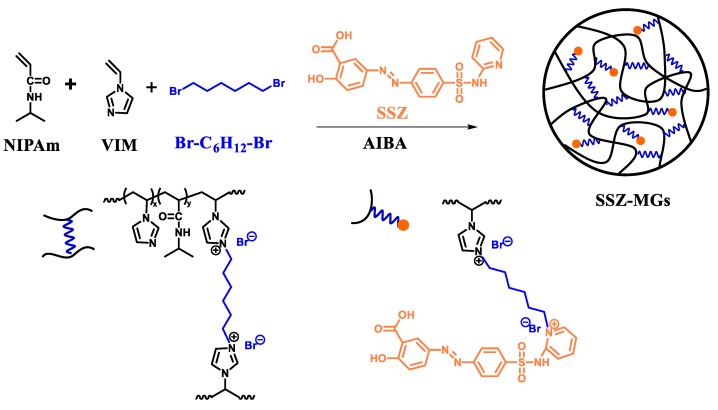
Synthesis route of SSZ functionalized microgels (SSZ-MGs) via quaternization reactions.

The sulfasalazine (SSZ) functionalized microgels (SSZ-MGs) were fabricated via in-situ quaternized crosslinking reaction during surfactant free emulsion polymerization (SFEP) of NIPAm and VIM in the presence of SSZ and Br-C_6_H_12_-Br with AIBA as the initiator, according to our method reported previously [22,23,30,31]. Control microgels without SSZ moieties were also fabricated by similar SFEP of NIPAm and VIM in the presence of Br-C_6_H_12_-Br, which were named as N-MGs. Scheme 1 shows the synthesis route of SSZ functionalized microgels. The detail synthesis conditions of SSZ-MGs were given in supporting information. 

### 2.3. Optical Detection of Fe^3+^ Ions in Aqueous Solution

The obtained SSZ-MGs were diluted with deionized water to a suitable concentration according to the absorption intensity of SSZ-MGs as determined by UV-vis spectroscopy. A volume of 20 μL freshly prepared aqueous solutions of metal ions with different concentrations were added into 2 mL diluted SSZ-MG aqueous suspensions. Deionized water with pH of 5.6 was used in all experiments except of otherwise stated. SSZ-MG aqueous suspensions with various pH values were obtained by adjusting the pH of suspensions with 1 M HCl and NaOH aqueous solutions. The pH values of SSZ-MG aqueous suspensions were measured by a pH meter (FE28, METTLER TOLEDO). The phosphate buffer solutions (PBS, 0.01 M, pH 5.62 and 7.0) and Tris-HCl (0.01 M, pH 7.1) buffer solution were prepared by the standard protocols.

### 2.4. Characterization

The morphology of SSZ-MGs was acquired on a JEOL JEM-1200 transmission electron microscopy (TEM) with 80 kV acceleration voltages. Dynamic light scattering (DLS) measurements of SSZ-MG aqueous suspensions were carried out by using a 90 Plus Particle Size Analyzer (Brookhaven Instruments Corp., Holtsville, NY, USA) with the laser wavelength λ of 635 nm. The samples were equilibrium at each temperature for 10 min before DLS measurements. UV-vis absorption spectra were obtained by using a Cary 100 instrument (Varian Australia Pty Ltd., Victoria, Australia) Note that a signal jump at ~350 nm would be observed in UV-vis absorption spectra because the Cary 100 instrument will automatically switch the light source at 350 nm during the data acquisition. The photographs of SSZ-MG aqueous suspensions were taken by using a SONY ILCE-6000L digital camera.

## 3. Results and Discussion

### 3.1. Synthesis and Characterization of SSZ-MGs

**Figure 1:**
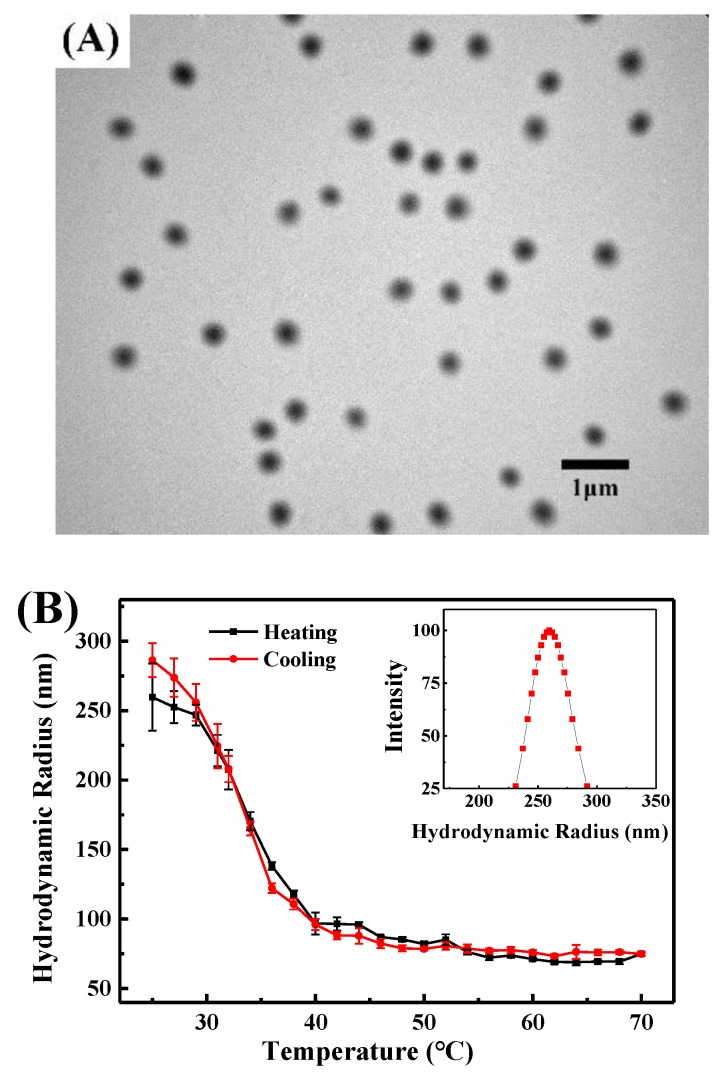
(**A**) Representative TEM image of SSZ-MGs and (**B**) hydrodynamic radius of SSZ-MGs in aqueous suspensions as a function of temperature measured by DLS. The inset of (**B**) showed the size distribution of SSZ-MGs in aqueous suspensions at 25 °C.

As shown in Scheme 1, the quaternization reaction between SSZ and Br-C_6_H_12_-Br, of which one Br end group has already quaternized the imidazole group of VIM, led to the incorporation of SSZ moieties in the microgel network. Furthermore, the quaternization reaction between VIM and Br-C_6_H_12_-Br formed the cross-linking network of resultant SSZ-MGs. Figure 1A shows the representative TEM image of obtained SSZ-MGs. Uniform SSZ-MGs with radius of about 205 ± 25 nm were observed. Figure 1B shows the hydrodynamic radius *R*_h_ of SSZ-MGs in aqueous suspensions as a function of temperature. A reversible thermosensitive behavior was observed for SSZ-MGs because of the polyNIPAm network chains. *R*_h_ of SSZ-MGs decreased with increasing temperature from 30 to 40 °C and reached a plateau value above 40 °C. Upon cooling the solution temperature, *R*_h_ of SSZ-MGs increased again along the similar track back to the original state within the experimental error. The size distribution of SSZ-MGs at 25 °C was shown in the inset of Figure 1B, which indicated the narrow size distribution of SSZ-MGs in aqueous suspensions. *R*_h_ of SSZ-MGs at 25 °C was about 259 ± 24 nm. From the first-order derivative of *R*_h_ versus temperature plot, the volume transition temperature (VTT) of SSZ-MGs in aqueous suspensions was determined to be about 34 °C, which indicated that SSZ-MGs became hydrophobic and collapsed at temperature above 34 °C. The value of 34 °C was smaller than those of 42–45 °C for P (NIPAm-co-VIM)/1,6-dibromohexane microgels, which were fabricated by SFEP of NIPAm and VIM in the presence of Br-C_6_H_12_-Br [30]. It was understandable that the incorporation of hydrophobic moieties SSZ into the microgel network would decrease the VTT of resultant microgels. 

**Figure 2:**
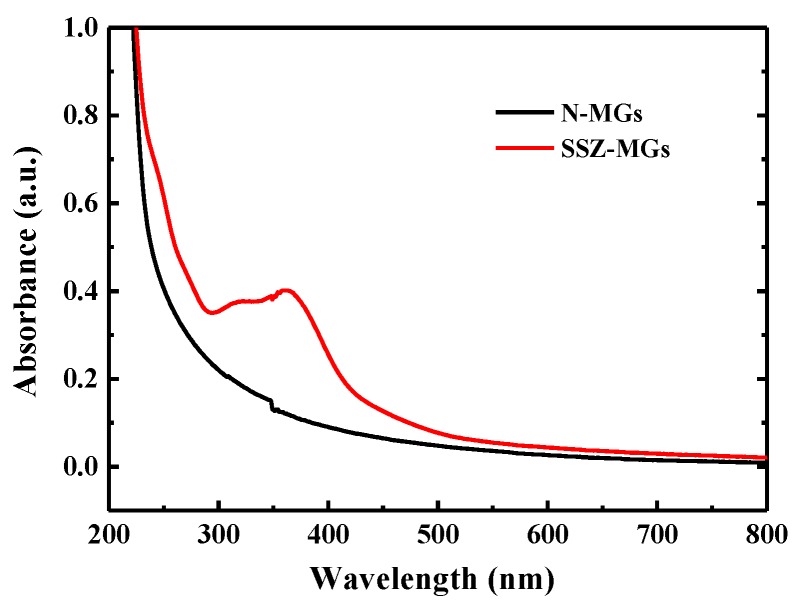
UV-vis spectra of N-MGs and SSZ-MGs aqueous suspensions with concentration of 0.099 mg/mL at 25 °C and pH of 5.6.

Figure 2 shows the UV-vis spectra of N-MG and SSZ-MG aqueous suspensions with concentration of 0.099 mg/mL. Clearly, a characteristic absorption peak at 362 nm was observed for SSZ-MG aqueous suspensions, which was assigned to the absorption of SSZ moieties. However, no absorption peak was observed for N-MG aqueous suspensions. The amount of SSZ moieties in SSZ-MGs was measured to be about 5.2 wt% by referring to the standard calibration curve of SSZ in DMF as shown in Figure S1, because SSZ cannot well dissolve in water. It was worthy to note that the characteristic absorption peak of SSZ bathochromic shifted to 405 nm in DMF because of the polarity of DMF. However, the use of the standard calibration curve of SSZ in DMF did not affect the calculation of SSZ contents in SSZ-MGs.

### 3.2. Optical Detection of Fe^3+^ Ions in Aqueous Solution

**Figure 3:**
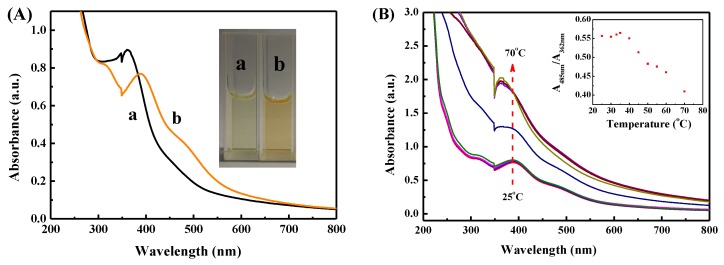

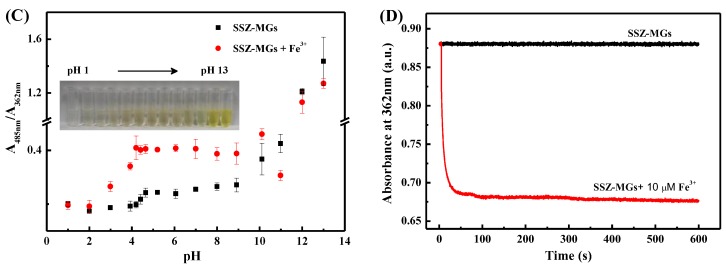
(**A**) UV-vis absorption spectrum of SSZ-MG aqueous suspensions (0.196 mg/mL) without (a) and with (b) the presence of 10 μM Fe^3+^ ions at 25 °C and pH of 5.6. The inset is the corresponding photographs of SSZ-MG aqueous suspensions without (a) and with (b) the presence of 10 μM Fe^3+^ ions. (**B**) UV-vis absorption spectra of SSZ-MG aqueous suspensions (0.196 mg/mL) with the presence of 10 μM Fe^3+^ ions at various temperatures and pH of 5.6. Inset shows the corresponding A_485nm_/A_362nm_ ratios. (**C**) The A_485nm_/A_362nm_ ratios of SSZ-MG aqueous suspensions (0.099 mg/mL) with and without the presence of 50 μM Fe^3+^ ions at 25 °C and various pH values. Inset of (**C**) shows the corresponding photographs of SSZ-MG aqueous suspensions with the presence of 50 μM Fe^3+^ ions. (**D**) Plot of absorption intensity at 362 nm of SSZ-MG aqueous suspensions (0.196 mg/mL) as a function of time after adding 10 μM Fe^3+^ ions at 25 °C and pH of 5.6.

Figure 3A shows the UV-vis absorption spectra of SSZ-MG aqueous suspensions without and with the presence of 10 μM Fe^3+^ ions at 25 °C and pH of 5.6. Note that the pH value of deionized water was about 5.6. The concentration of SSZ-MG aqueous suspensions was 0.196 mg/mL. It can be seen from Figure 3A that the original SSZ-MG aqueous suspensions showed a light-yellow color and a characteristic absorption peak at 362 nm. However, with the presence of 10 μM Fe^3+^ ions, the color of SSZ-MG aqueous suspensions changed to orange, and the characteristic absorption peak at 362 nm was bathochromic shift to 388 nm, accompanied with the appearance of a new shoulder peak at 485 nm. These results indicated that SSZ-MGs can respond to Fe^3+^ ions in aqueous solution. 

Since SSZ-MGs exhibited thermosensitive character, we studied the response of SSZ-MG aqueous suspensions to Fe^3+^ ions at various temperatures, as shown in Figure 3B. The SSZ-MG aqueous suspensions were first equilibrium at each temperature (pH of 5.6) for 10 min before addition of 10 μM Fe^3+^ ions. It can be seen from Figure 3B that the overall absorption intensity increased with increasing temperature because the SSZ-MGs shrunk, and the microgel suspension became turbid with increasing temperature. The inset of Figure 3B shows the intensity ratio A_485nm_/A_362nm_ of the characteristic absorption peaks at 485 nm and 362 nm as a function of temperature. When the temperature was above 35 °C, the A_485nm_/A_362nm_ ratios of SSZ-MG aqueous suspensions with the presence of 10 μM Fe^3+^ ions began to decrease. It was understandable because Fe^3+^ ions were more difficult to diffuse into the shrunk microgels and interact with the SSZ moieties at higher temperature, leading to the relative decrease of absorption intensity at 485 nm. The UV-vis absorption spectra of SSZ-MG aqueous suspensions without the presence of Fe^3+^ ions at various temperatures were shown in Figure S2. Similarly, the overall absorption intensity of SSZ-MG aqueous suspensions increased with increasing temperature due to the shrinking and collapse of SSZ-MGs. However, the A_485nm_/A_362nm_ ratio of SSZ-MG aqueous suspensions without the presence of Fe^3+^ ions increased from 0.25 to 0.43 (inset of Figure S2), which were attributed to the fact that the absorption intensity at longer wavelength of 485 nm increased more than that at 362 nm although there was not absorption peak at 485 nm.

The effects of pH value on the response of SSZ-MG aqueous suspensions to Fe^3+^ were further investigated, as shown in Figure 3C. It can be seen from Figure 3C that the A_485nm_/A_362nm_ values of SSZ-MG aqueous suspensions (0.099 mg/mL) with the presence of 50 μM Fe^3+^ ions at pH of 1 and 2 were similar with those of original SSZ-MG aqueous suspensions without the presence of Fe^3+^ ions. The UV-vis absorption spectra of SSZ-MG aqueous suspensions with or without the presence of 50 μM Fe^3+^ ions were almost the same as shown in Figure S3A, which indicated that SSZ-MGs did not respond to Fe^3+^ at pH 1 and 2. For the pH range of 4.6 to 9, the A_485nm_/A_362nm_ values of SSZ-MG aqueous suspensions with or without the presence of 50 μM Fe^3+^ ions were unaffected by pH of the suspensions. However, for pH above 10, the UV-vis absorption spectra of SSZ-MG aqueous suspensions were completely different with those at lower pH values, as shown in Figure S3B. Abroad strong absorption peak appeared at 452 nm regardless of the presence of Fe^3+^ ions or not, which might be due to the ionized form of SH_3_^3−^ at strong alkaline conditions with pH of 12 and 13. Similar results were observed for SSZ-MG aqueous suspensions (0.099 mg/mL) with the presence of 10 μM Fe^3+^ ions at various pH values, as shown in Figure S3C. Different colors of SSZ-MG aqueous suspensions with the presence of 50 μM Fe^3+^ ions at low pH (1 and 2) and high pH (12 and 13) were clearly observed as shown in the inset of Figure 3C. It had been reported that there were four kinds of molecular forms of sulfasalazine (SSZ) molecule in aqueous solution depending on the pH value [32]: the neutral non-ionized form (SH^0^); the ionized form with deprotonation of carboxyl group (SH^−^, pK_a1_ = 2.9); the ionized form with deprotonation of both carboxyl and phenolic group (SH_2_^2−^, pK_a1_ = 8.7); and the ionized form with deprotonation of carboxyl, phenolic, and sulfonamide group (SH_3_^3−^, pK_a1_ = 11.1), as shown in Figure S4. Therefore, the SSZ moieties of SSZ-MGs exhibited the ionized form of SH^−^ and SH_2_^2−^ at pH of 5.6. Furthermore, the results of Figure 3C also suggested that the SSZ-MGs with SH^0^ and SH_3_^3−^ molecular forms of SSZ moieties did not lead to observable and distinguishable response to Fe^3+^ ions in aqueous solution. The effect of pH value on the hydrodynamic radius of SSZ-MG aqueous suspensions were also studied, as shown in Figure S5. The results of Figure S5 indicated that the hydrodynamic radius of SSZ-MG aqueous suspensions was almost unaffected in the pH range of 4.6 to 9. 

Figure 3D shows the response kinetic of SSZ-MG aqueous suspensions to Fe^3+^ ions. It can be seen that the absorption intensity at 362 nm of SSZ-MG aqueous suspensions strongly decreased right after the addition of 10 μM Fe^3+^ ions. After about 120 s, the absorption intensity at 362 nm reached a plateau value. For pure SSZ-MG aqueous suspensions, the absorption intensity at 362 nm kept constant with time. This result indicated that it took about 120 s for Fe^3+^ to diffuse and coordinate with the SSZ moieties of SSZ-MGs. According to the above experimental results, 25 °C and pH of 5.6 were chosen as the optimum conditions for the optical detection of Fe^3+^ ions in aqueous solution. Furthermore, for the sake of diffusion equilibrium, the time for recording the UV-vis absorption spectrum of SSZ-MG aqueous suspensions after the addition of Fe^3+^ ions was taken as 200 s.

**Figure 4:**
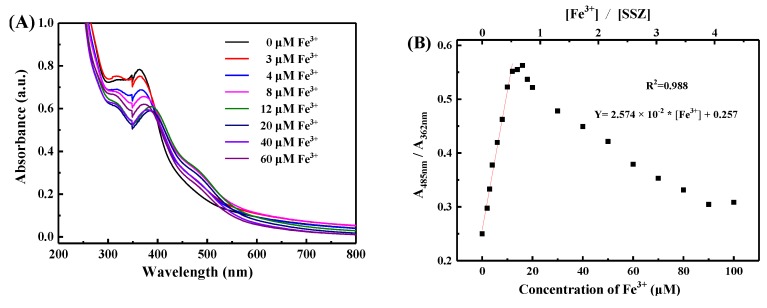

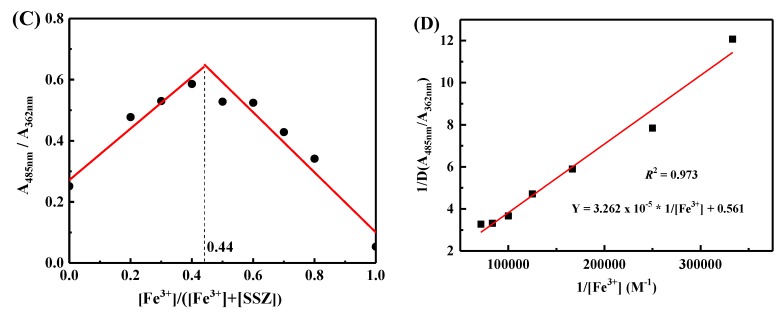
(**A**) Representative UV-vis absorption spectra of SSZ-MG aqueous suspensions with the presence of various concentrations of Fe^3+^ ions at 25 °C and pH of 5.6. The concentration of SSZ-MG aqueous suspensions was 0.174 mg/mL. The corresponding concentration of SSZ moieties ([SSZ]) in SSZ-MG aqueous suspensions was 23.09 μM. (**B**) A_485_nm/A_362_nm ratio as a function of Fe^3+^ concentration ([Fe^3+^]) as well as [Fe^3+^]/[SSZ] ratio. (**C**) Job’s plot of SSZ-MGs and Fe^3+^. The total concentration ([SSZ] + [Fe^3+^]) of SSZ moieties in SSZ-MGs and Fe^3+^ ions was kept constant to be 20 μM. (**D**) Modified Benesi–Hilderbrand plot of SSZ-MGs with Fe^3+^ in the [Fe^3+^] concentration range of 3–14 μM.

Figure 4A shows the representative UV-vis adsorption spectra of SSZ-MG aqueous suspensions with various Fe^3+^ concentrations at 25 °C and pH of 5.6. It can be seen that with increasing the concentration of Fe^3+^ ions in the range of 0–16 μM, the absorption intensity at 485 nm increased and the absorption intensity at 362 nm decreased. Furthermore, the absorption peak at 362 nm slowly red shifted to 388 nm with increase in the concentration of Fe^3+^ ions, as shown in Figure S6. With increase in the concentration of Fe^3+^ ions, the intensity ratio of A_485nm_/A_362nm_ first increased linearly in the range of 0–12 μM and reached the value of about 0.56 at 16 μM and then slowly decreased to 0.30 at 100 μM, as shown in Figure 4B. A linear relationship between the A_485nm_/A_362nm_ ratios and the concentrations of Fe^3+^ ions was found to be Y = 2.574 × 10^−2^ * [Fe^3+^] + 0.257 with R^2^ value of 0.988 for the concentration of Fe^3+^ ions in the range of 0–12 μM. The limit of detection (D_L_) for SSZ-MG aqueous suspension in detection of Fe^3+^ ions in aqueous solution could be then determined to be about 0.110 μΜ (0.006 mg/L) from this linear relationship according to the 3α IUPAC criteria [23,33]. D_L_ of 0.110 μΜ (0.006 mg/L) was about 50 times lower than the maximum permitted level of Fe^3+^ (0.3 mg/L, 5.4 μΜ) in drinking water by EPA of the United States. Figure 4B also shows that the A_485nm_/A_362nm_ ratio first increased with increasing the concentration of Fe^3+^ ions [Fe^3+^] and reached a maximum value when the [Fe^3+^]/[SSZ] ratio reached about 0.7. Note that [Fe^3+^]/[SSZ] was the ratio of molar concentration of Fe^3+^ ions to SSZ moieties in SSZ-MG aqueous suspensions. The SSZ moieties were gradually chelating with Fe^3+^ ions with increase in the concentration of Fe^3+^ ions, and the A_485nm_/A_362nm_ ratio reached the maximum at coordination equilibrium state. Further increasing Fe^3+^ concentrations, the values of A_485nm_/A_362nm_ ratio decreased. It is also worthy to note that the concentration of SSZ-MG aqueous suspensions did not affect the coordination behavior between Fe^3+^ and SSZ moieties of SSZ-MGs, as shown in Figure S7. As discussed above, the SSZ moieties of SSZ-MGs adopted the ionized form of SH^−^ in aqueous suspensions at pH of 5.6. The deprotonation of carboxyl group (SH^−^, pK_a1_ = 2.9) and non-ionized phenolic group might coordinate with Fe^3+^ ions in the solution. Figure S8 shows the possible coordination structures of SSZ moieties and Fe^3+^ ion. Accordingly, one Fe^3+^ ion can complex with one or two SSZ moieties, leading to the possible [Fe^3+^]/[SSZ] ratio of 1 or 0.5, which was consistent with the results obtained from Figure 4B and Figure S7. The binding stoichiometry of SSZ-MGs with Fe^3+^ was further determined by Job’s plot [34,35], as shown in Figure 4C. The obtained Job’s plot showed that the A_485nm_/A_362nm_ ratio approached a maximum value when the molar ratio of [Fe^3+^]/([Fe^3+^] + [SSZ]) was about 0.44. This result indicated that one Fe^3+^ ion mostly complexed with one SSZ moiety, i.e., 1:1 stoichiometry for the complex between SSZ moiety and Fe^3+^. At the same time, there were also the complexes of one Fe^3+^ ion with two SSZ moieties, i.e., 2:1 stoichiometry for the complex between SSZ moiety and Fe^3+^. Since the binding stoichiometry of SSZ moiety with Fe^3+^ was close to 1:1, the apparent association constants *K*_a_ of complex of SSZ moiety with Fe^3+^ could be also estimated by a modified Benesi–Hilderbrand equation given as [36,37]:

1/Δ(A_485nm_/A_362nm_) = 1/Δ(A_485nm_/A_362nm_)_sat_ + 1/(Δ(A_485nm_/A_362nm_)_sat_*K*_a_[Fe^3+^]) (1)

where Δ(A_485nm_/A_362nm_) is the difference of A_485nm_/A_362nm_ ratio between the apparent metal complex and the free SSZ-MGs, and Δ(A_485nm_/A_362nm_)_sat_ is the different A_485nm_/A_362nm_ ratio at saturation. The association constant *K*_a_ was evaluated to be 1.72 × 10^4^ M^−1^ (*R*^2^ = 0.973) from the plot of 1/Δ(A_485nm_/A_362nm_) versus 1/[Fe^3+^] in the concentration range of 3–14 μM, as shown in Figure 4D. 

It might be argued that the addition of Fe^3+^ ions will change the pH of SSZ-MG aqueous suspensions and hence affect the sensitivity of Fe^3+^ ion detection. The pH values of SSZ-MG aqueous suspensions as a function of time were monitored immediately after addition of 2, 10, 15 and 30 μM Fe^3+^ ions at 25 °C, as shown in Figure S9A. It can be seen that the pH value of SSZ-MG aqueous suspensions dropped from 5.6 to about 5.0 after addition of 2 μM Fe^3+^ ions and then maintained the pH value in the measured period of 10 min. For addition of 10 and 15 μM Fe^3+^ ions, the pH value of SSZ-MG aqueous suspensions dropped from 5.6 to an equilibrium value of 4.6 in the measured period of 10 min. However, for addition of 30 μM Fe^3+^ ions, the pH of SSZ-MG aqueous suspensions decreased immediately from 5.6 to 4.6 and then slowly to 4.2 within 5 min and maintained the pH of 4.2 up to the measured time of 10 min. The pH values of SSZ-MG aqueous suspensions after addition of 2, 10 and 15 μM Fe^3+^ ions were in the range of 4.6–9, which justified the linear concentration range of 0–16 μM for the detection of Fe^3+^ ions. According to the results of Figure 3C, A_485nm_/A_362nm_ ratio was unaffected by the pH value in such pH range. Figure S9B also shows the hydrodynamic radius of SSZ-MG aqueous suspensions as a function of time after addition of 10 μM Fe^3+^ ions. It can be seen that the hydrodynamic radius of SSZ-MGs slightly increased with addition of Fe^3+^ ions. Possibly, the coordination of Fe^3+^ and hydrophobic SSZ moieties made the SSZ-MGs more hydrophilic, hence leading to the swelling of SSZ-MGs and the increase of hydrodynamic size. Furthermore, the detection sensitivity of SSZ-MGs in buffer solutions was also investigated. The original SSZ-MG aqueous suspensions were diluted by using PBS and Tris-HCl buffer solutions, respectively. It was found that the A_485nm_/A_362nm_ ratios of SSZ-MG PBS buffer suspensions with pH values of 5.6 and 7.0 after addition of 10 μM Fe^3+^ ions were the same as that of original value (0.21) before addition of Fe^3+^ ions, indicating that the SSZ-MG PBS buffer suspensions lost the response to Fe^3+^ ions. These results suggested that the phosphate ions might deteriorate the coordination of SSZ moieties and Fe^3+^ ions. However, SSZ-MG Tris-HCl buffer suspensions can respond to Fe^3+^ ions, and a similar response behavior to those in Figure 4B was observed. Figure S10A shows that the A_485nm_/A_362nm_ ratio of SSZ-MG Tris-HCl buffer suspensions first increased with increasing [Fe^3+^] and reached a maximum value when the [Fe^3+^]/[SSZ] ratio reached about 0.75, which was close to the value of 0.7 for SSZ-MG aqueous suspensions (cf. Figure 4B). Similarly, a linear relationship between the A_485nm_/A_362nm_ ratios and the concentrations of Fe^3+^ ions was found to be Y = 1.316 × 10^−2^ × [Fe^3+^] + 0.2 with *R^2^* value of 0.986 for the concentration of Fe^3+^ ions in the range of 0–12 μM, which indicated that the SSZ-MG Tris-HCl buffer suspensions had less sensitivity toward Fe^3+^ ions compared with those results of SSZ-MG aqueous suspension (cf. Figure 4B). Furthermore, Figure S10B shows that the response time of SSZ-MG Tris-HCl buffer suspensions toward Fe^3+^ ions was also less than 200 s. The reason for the decrease of A_485nm_/A_362nm_ ratios at higher [Fe^3+^] above the maximum coordination concentration was unclear. Possibly, the excess Fe^3+^ ions might result in the partial hydrolysis of Fe^3+^ ions, generating Fe(OH)_2_ and potent oxidants such as HO·and $O_{2}^{-}$ [38]. The HO and $O_{2}^{-}$ were strong coordination ligands, which might in turn compromise the interaction between Fe^3+^ and SSZ moieties, leading to the decrease of A_485nm_/A_362nm_ ratio as observed in Figure 4B and Figure S10A.

### 3.3. Selectivity and Interference Study over Other Metal Ions 

**Figure 5:**
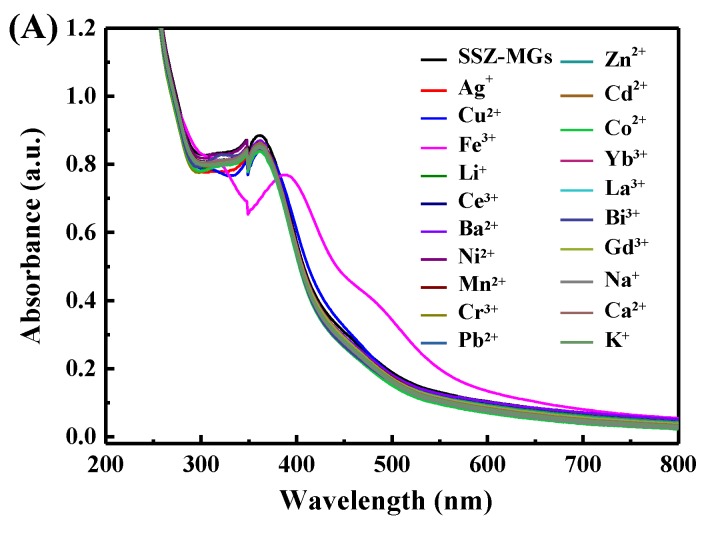

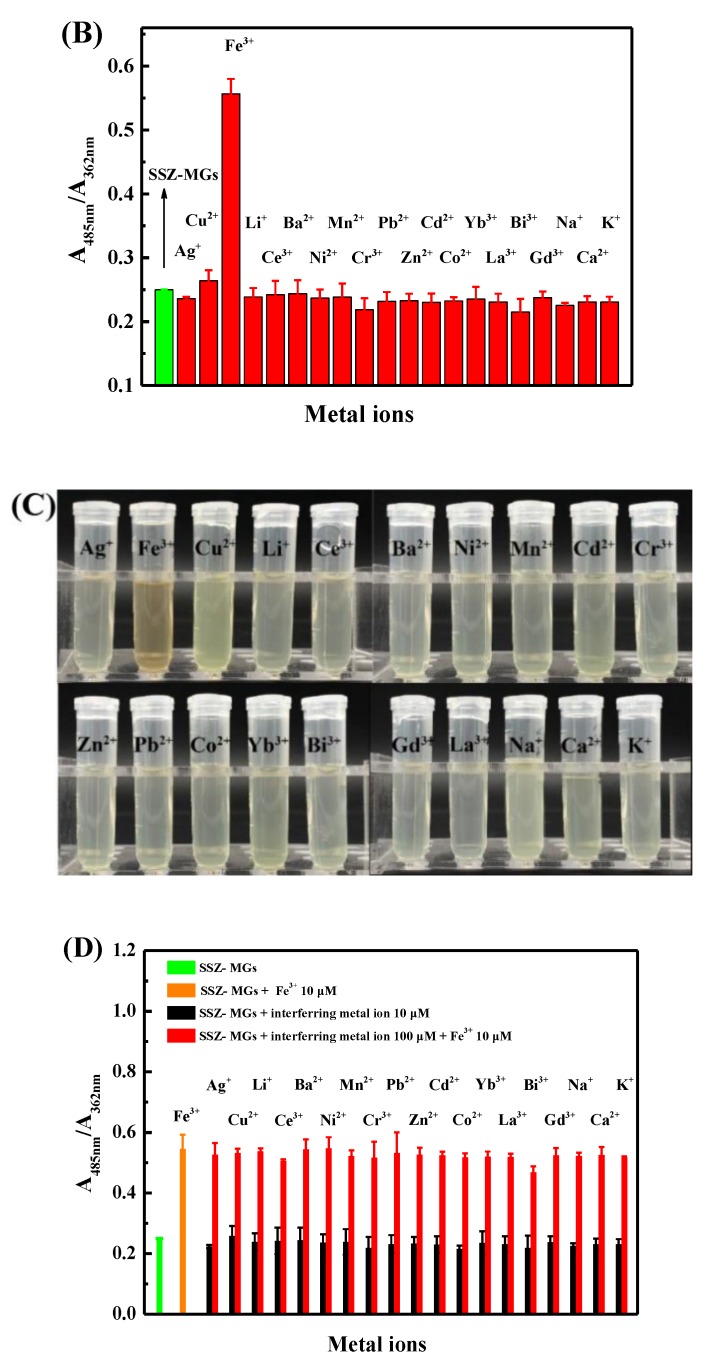
(**A**) UV-vis absorption spectra of SSZ-MG aqeuous suspensions (0.196 mg/mL) at 25 °C and pH of 5.6 with the presence of 10 μM different metal ions, respectively. (**B**) The corresponding A_485nm_/A_362nm_ ratios calculated from the spectra shown in (A). (**C**) The photographs of SSZ-MG aqueous suspensions (0.196 mg/mL) after addition of 10 μM various metal ions, respectively. (**D**) The interference studies of different metal ions (100 μM) on the response of SSZ-MG aqueous suspensions (0.196 mg/mL) to Fe^3+^ ions (10 μM) at 25 °C and pH of 5.6.

The interference of other metal ions and selectivity of SSZ-MGs for the detection of Fe^3+^ ions in aqueous solution were further investigated. Figure 5A shows the UV-vis absorption spectra of SSZ-MG aqueous suspensions at 25 °C and pH of 5.6 after addition of 10 μM of various metal ions, namely, Ag^+^, Li^+^, Na^+^, K^+^, Ca^2+^, Ba^2+^, Cu^2+^, Ni^2+^, Mn^2+^, Pb^2+^, Zn^2+^, Cd^2+^, Co^2+^, Cr^3+^, Yb^3+^, La^3+^, Gd^3+^, Ce^3+^, Bi^3+^ and Fe^3+^, respectively. Clearly, the addition of 10 μM Fe^3+^ ions resulted in a significant change of the absorption spectrum of SSZ-MG aqueous suspensions. The addition of 10 μM Cu^2+^ ions resulted in the red shift of absorption spectrum but less significant than that caused by Fe^3+^ ions. Furthermore, no shoulder absorption peak was observed at 485 nm. By adding other metal ions, the shape of absorption spectra was generally unchanged, but the overall absorption intensity slightly decreased. Figure 5B shows that A_485nm_/A_362nm_ ratio significantly increased after addition of Fe^3+^ ions. With addition of Cu^2+^ ions, the A_485nm_/A_362nm_ ratio only slightly increased. By adding the rest of other metal ions, the A_485nm_/A_362nm_ ratio slightly decreased as compared to the value of pure SSZ-MG aqueous suspensions. Figure 5C shows the photographs of SSZ-MG aqueous suspensions after addition of 10 μM various metal ions. The color of SSZ-MG aqueous suspensions with the presence of 10 μM Fe^3+^ ions was strongly different to those with the presence of other metal ions and can be clearly identified by the naked eye. These results indicated that the SSZ-MGs can selectively respond to Fe^3+^ ions in aqueous solutions. Note that the N-MG aqueous suspensions showed no response to the 20 species of metal ions studied here, and the SSZ molecules in DMF cannot selectively respond to Fe^3+^ ions, as shown in Figure S11. Figure 5D shows the A_485nm_/A_362nm_ ratios of SSZ-MG aqueous suspensions containing 10 μM Fe^3+^ ions when adding various interfering metal ions with concentrations of 100 μM, at 25 °C and pH of 5.6. It can be seen from Figure 5D that the presence of other metal ions (19 species studied here) with concentration even 10 times higher than that of Fe^3+^ ions did not interfere with the response of SSZ-MG aqueous suspensions to Fe^3+^ ions in aqueous solution. Figure S12 shows the variation of A_485nm_/A_362nm_ ratios for SSZ-MG aqueous suspensions and SSZ-MG Tris-HCl buffer suspensions by sequentially adding 10 μM various metal ions. It can be seen that for the SSZ-MG aqueous suspensions, the previous added metal ions did not affect selective response of SSZ-MG aqueous suspensions to Fe^3+^ ions. The final addition of Fe^3+^ ions resulted in a significant increase of A_485nm_/A_362nm_ ratio, as shown in Figure S12A. However, for SSZ-MG Tris-HCl buffer suspensions, the existence of other metal ions did affect the response of SSZ-MGs to certain extent. Figure S12B indicates that SSZ-MG Tris-HCl buffer suspensions exhibited less selectivity toward Fe^3+^ ions over other metal ions. These results indicated and justified that SSZ-MG aqueous suspensions are suitable to highly selective and sensitive detect micromole Fe^3+^ ions in aqueous solution. 

The potential application of SSZ-MG aqueous suspensions as optical sensor to detect trace Fe^3+^ ions in daily drinking water and lake water taken from Yuquan campus of Zhejiang University was preliminary tested, as shown in S1. Deionized water was also tested. Since the original concentrations of Fe^3+^ ions in the three water samples were less than the D_L_ of SSZ-MG aqueous suspensions as determined by elemental analysis, arbitrary amounts of Fe^3+^ ions were then spiked into the water samples before measurement. The results of Table S1 show that the concentration of Fe^3+^ ions spiked in the three water samples studied here can be accurately determined within the experimental error by using SSZ-MG aqueous suspensions as the optical sensor.

## 4. Conclusions

Sulfasalazine (SSZ) functionalized microgels (SSZ-MGs) with hydrodynamic radius of about 259 ± 24 nm and uniform size distribution at 25 °C were successfully synthesized via in-situ quaternization reaction. The SSZ-MG aqueous suspensions can optically respond to trace Fe^3+^ ions in aqueous solution within 200 s at 25 °C and pH of 5.6 with high sensitivity and selectivity over other 19 species of metal ions. A linear concentration range of 0–12 μM was found for the detection of Fe^3+^ ions in aqueous solution by using SSZ-MG aqueous suspensions at 25 °C and pH of 5.6. The limit of detection was about 0.110 μM (0.006 mg/L) Fe^3+^ ions in aqueous solution, which was much lower than the maximum allowance level of Fe^3+^ ions in drinking water (0.3 mg/L) regulated by EPA of the United States. Such SSZ-MG aqueous suspensions might have potential application as optical sensors to detect trace Fe^3+^ ions in real world samples.

## Supplementary Materials

The following are available online at https://www.mdpi.com/1424-8220/19/19/4223/s1. Figure S1: (A) UV-vis spectra of SSZ in DMF with various concentrations at room temperature. (B) The corresponding standard calibration curve of SSZ in DMF. Figure S2: The UV-vis absorption spectra of SSZ-MG aqueous suspensions without the presence of Fe^3+^ ions at various temperatures. Inset shows the corresponding A_485nm_/A_362nm_ ratios. The concentration of SSZ-MG aqueous suspensions was 0.196 mg/mL. Figure S3: The UV-vis absorption spectra of SSZ-MG aqueous suspensions (0.099 mg/mL) with or without the presence of 50 μM Fe^3+^ ions at (A) pH 1 and 2, and (B) pH 12 and 13. (C) The A_485nm_/A_362nm_ ratios of SSZ-MG aqueous suspensions (0.099 mg/mL) with and without the presence of 10 μM Fe^3+^ ions at 25 °C and various pH values. Figure S4: Possible structures of sulfasalazine (SSZ) moieties in SSZ-MG aqueous suspensions. Figure S5: The hydrodynamic radius of SSZ-MG aqueous suspensions as a function of pH values at 25 °C, which were adjusted by using 1 M HCl and NaOH aqueous solutions. The red circle symbol presented the hydrodynamic radius of original diluted SSZ-MG aqueous suspensions without pH adjusting. Figure S6: Wavelength shift of absorption peak at 362 nm of SSZ-MG aqueous suspensions as a function of Fe^3+^ concentration. Figure S7: A_485nm_/A_362nm_ ratios as a function of [Fe^3+^]/[SSZ] for SSZ-MG aqueous suspensions with various concentrations at 25 °C and pH of 5.6. The concentrations of SSZ-MG aqueous suspensions were 0.099, 0.082, and 0.075 mg/mL, respectively. The corresponding concentrations of SSZ moieties were 13.19, 10.88, and 9.95 μM, respectively. Figure S8: Possible coordination structures of SSZ moieties and Fe^3+^ ion. Figure S9: (A) The pH values of SSZ-MG aqueous suspensions (0.174 mg/mL) as a function of time monitored immediately after addition of 2, 10, 15, and 30 μM Fe^3+^ ions at 25 °C. (B) The hydrodynamic radius of SSZ-MG aqueous suspensions as a function of time measured by DLS immediately after addition of 10 μM Fe^3+^ ions at 25 °C. The red symbol was the hydrodynamic radius of original SSZ-MG aqueous suspensions. Figure S10: (A) The A_485nm_/A_362nm_ ratio of SSZ-MG Tris-HCl buffer suspensions (0.196 mg/mL) as a function of Fe^3+^ concentration ([Fe^3+^]) as well as [Fe^3+^]/[SSZ] ratio. (B) Plot of absorption intensity at 362 nm of SSZ-MG Tris-HCl buffer suspensions (0.196 mg/mL) as a function of time after adding 10 and 20 μM Fe^3+^ ions. Figure S11: (A) The UV-vis absorption spectra of N-MG aqueous suspensions (0.099 mg/mL) after addition of 50 μM various metal ions, respectively. (B) The UV-vis absorption spectra of SSZ molecules in DMF (0.012 mg/mL) with the presence of 50 μM various metal ions, respectively. Figure S12: (A) The A_485nm_/A_362nm_ ratios of SSZ-MG aqueous suspensions (0.196 mg/mL, pH 5.6) upon sequential addition of different metal ions (10 μM) at 25 °C. (B) The A_485nm_/A_362nm_ ratios of SSZ-MG Tris-HCl buffer suspensions (0.196 mg/mL, pH 7.1) upon sequential addition of different metal ions (10 μM) at 25 °C. The addition sequences of metal ions were Ag^+^, Li^+^, Ce^3+^, Ba^3+^, Ni^2+^, Mn^2+^, Cr^3+^, Pb^2+^, Zn^2+^, Cd^2+^, Co^2+^, Yb^3+^, La^3+^, Gd^3+^, Na^+^, K^+^, Ca^2+^, Cu^2+^, Bi^3+^ and Fe^3+^ for (A) and (B). Table S1: Concentrations of Fe^3+^ ions spiked in deionized water, lake water, and drinking water from Yuquan campus of Zhejiang University as determined by UV-vis absorption spectroscopy with SSZ-MG aqueous suspensions (0.082 mg/mL) as the optical sensor.
