# Supplementary material for: Optical Detection of Fe3+ Ions in Aqueous Solution with High Selectivity and Sensitivity by Using Sulfasalazine Functionalized Microgels
Source: Sensors (Basel). 2019 Sep 28;19(19):4223. doi: 10.3390/s19194223 (PMC6806204; doi:10.3390/s19194223)
Supplement: Supplementary file 1 [file sensors-19-04223-s001.pdf]

Supplementary Materials:

Optical Detection of Fe^{3+} Ions in Aqueous Solution with High Selectivity and Sensitivity by Using Sulfasalazine Functionalized Microgels

Weiming Ji [1], Zumei Zhu [2], Shunni Dong [1], Jingjing Nie [2] and Binyang Du [1,*]

[†] MOE Key Laboratory of Macromolecular Synthesis and Functionalization, Department of Polymer Science & Engineering, Zhejiang University, Hangzhou 310027, China

[‡] Department of Chemistry, Zhejiang University, Hangzhou 310027 and China

[*]Corresponding author. E-mail: duby@zju.edu.cn.

Additional Experimental Section

1. Materials

Gadolinium(III) nitrate hexahydrate ($Gd(NO_3)_3{\cdot}6H_2O$, 99.9%), lithium nitrate ($LiNO_3$, 99%), cerium(III) nitrate hexahydrate ($Ce(NO_3)_3{\cdot}6H_2O$, 99%), manganese(II) nitrate tetrahydrate ($Mn(NO_3)_2{\cdot}4H_2O$, 97.5%), bismuth(III) nitrate pentahydrate ($Bi(NO_3)_3{\cdot}5H_2O$, 98%), ytterbium(III) nitrate pentahydrate ($Yb(NO_3)_3{\cdot}5H_2O$, 99.99%), cadmium nitrate tetrahydrate ($Cd(NO_3)_2{\cdot}4H_2O$, 98%), and lanthanum(III) nitrate hydrate ($La(NO_3)_3{\cdot}H_2O$, 99%), were obtained from J&K Chemical Ltd. Chromium(III) nitrate nonahydrate ($Cr(NO_3)_3{\cdot}9H_2O$, 99%) was obtained from Shanghai Aladdin Bio-Chem Technology Co., Ltd. Potassium nitrate (KNO_3, 99%), silver nitrate ($AgNO_3$, 99.8%), sodium nitrate ($NaNO_3$, 99%), iron(III) nitrate nonahydrate ($Fe(NO_3)_3{\cdot}9H_2O$, 98.5%), cobalt(II) nitrate hexahydrate ($Co(NO_3)_2{\cdot}6H_2O$, 99%), copper(II) nitrate trihydrate ($Cu(NO_3)_2{\cdot}3H_2O$, 99.5%), lead(II) dinitrate ($Pb(NO_3)_2$, 99%), zinc nitrate hexahydrate ($Zn(NO_3)_2{\cdot}6H_2O$, 99%), barium nitrate ($Ba(NO_3)_2$, 99.5%), nickel(II) nitrate hexahydrate ($Ni(NO_3)_2{\cdot}6H_2O$, 98%), and calcium nitrate tetrahydrate ($Ca(NO_3)_2{\cdot}4H_2O$, 98.5%) were obtained from Sinopharm Chemical Reagent Co., Ltd.

2. Synthesis Procedure of Sulfasalazine Functionalized Microgels (SSZ-MGs)

For the synthesis of SSZ-MGs, NIPAm (226.4 mg, 2 mmol), VIM (27 μL, 0.3 mmol), and 1,6-dibromhexane (30 μL, 0.2 mmol) were added into deionized water (50 mL) in a 100 mL three-neck flask. The mixture was then heated to 70 °C with vigorous stirring and bubbled with N_2 for 30 min. After that, 1 mL of AIBA aqueous solution (25 mg/mL) was added to the mixture to initiate the polymerization reaction. After 1 h, 1 mL of SSZ DMF solution (78.8 mg, 0.2 mmol) was added drop wise to the reaction mixture. The reaction further proceeded at 70 °C for 24 h. After polymerization, the reaction mixture was cooled down to room temperature. The reaction mixture was first dialyzed against DMF for 2 days and then deionized water for 7 days. The dialysis tubes with MWCO of 14000 were used. During dialysis, DMF or deionized water was changed every 12 h. The obtained purified microgels were named as SSZ-MGs. The yield of SSZ-MGs was about 50.3%.

N-MGs were synthesized by the same procedure without addition of SSZ molecules.

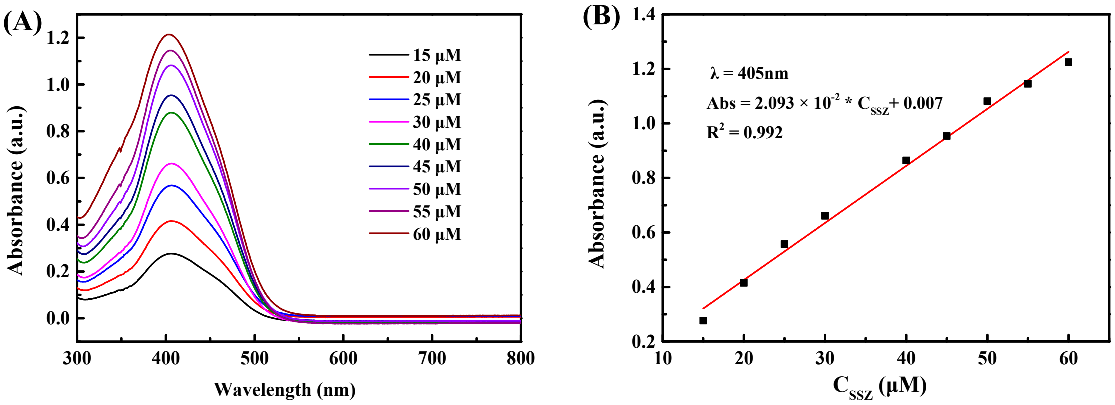

Figure S1. (**A**) UV-vis spectra of SSZ in DMF with various concentrations at room temperature. (**B**) The corresponding standard calibration curve of SSZ in DMF.

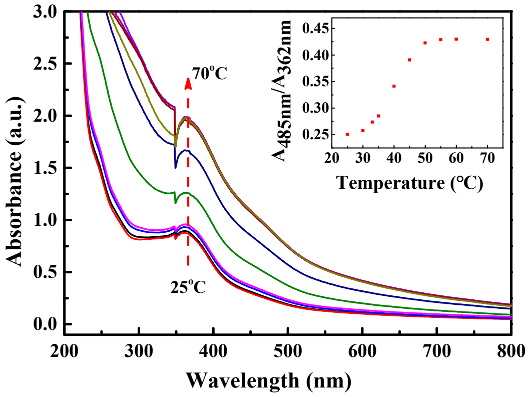

Figure S2. The UV-vis absorption spectra of SSZ-MG aqueous suspensions without the presence of Fe^{3+} ions at pH of 5.6 and various temperatures. Inset shows the corresponding A_{485nm}/A_{362nm} ratios. The concentration of SSZ-MG aqueous suspensions was 0.196 mg/mL.

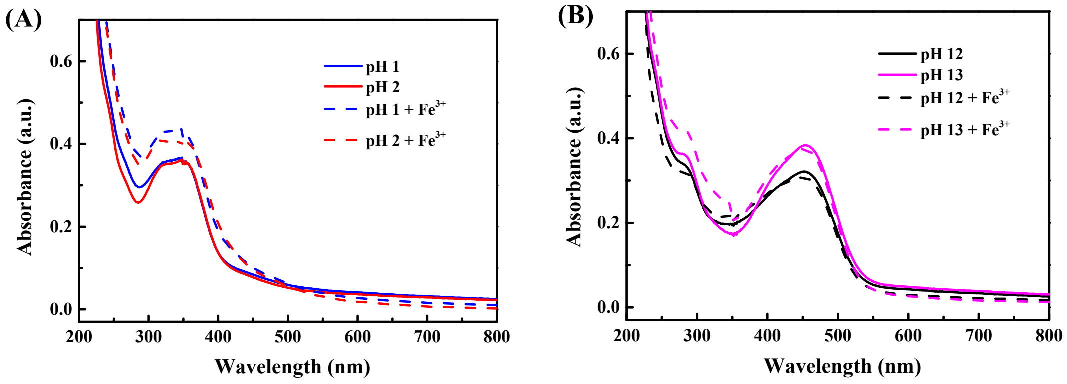

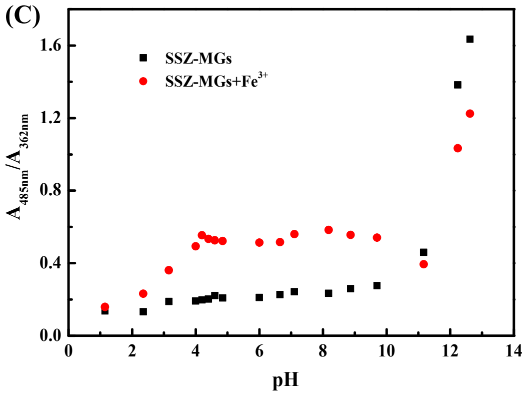

Figure S3. The UV-vis absorption spectra of SSZ-MG aqueous suspensions (0.099 mg/mL) with or without the presence of 50 μM Fe^{3+} ions at (**A**) pH 1 and 2, and (**B**) pH 12 and 13. (**C**) The A_{485nm}/A_{362nm} ratios of SSZ-MG aqueous suspensions (0.099 mg/mL) with and without the presence of 10 μM Fe^{3+} ions at 25 °C and various pH values.

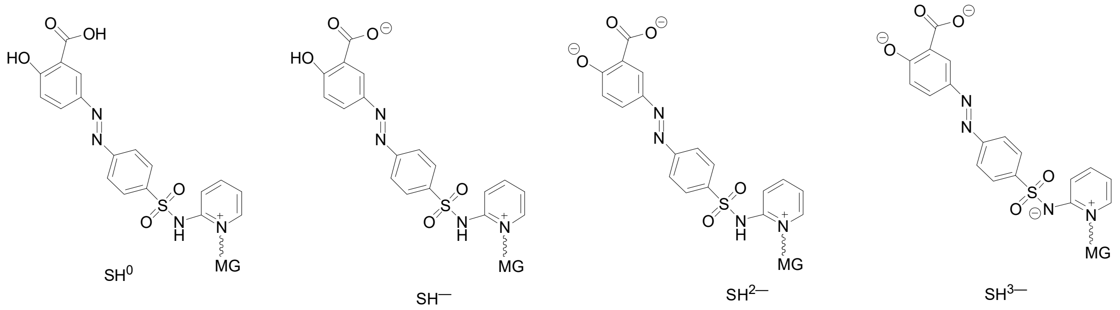

Figure S4. Possible structures of sulfasalazine (SSZ) moieties in SSZ-MG aqueous suspensions.

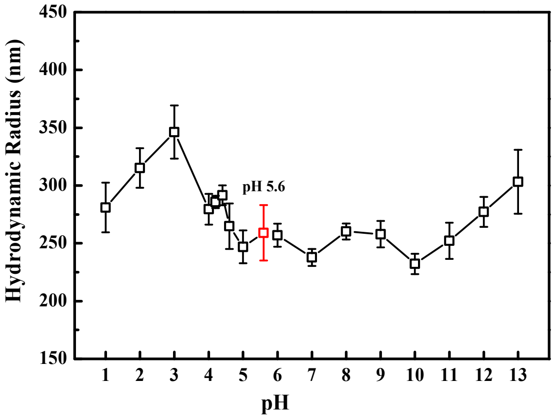

Figure S5. The hydrodynamic radius of SSZ-MG aqueous suspensions as a function of pH values at 25 °C, which were adjusted by using 1 M HCl and NaOH aqueous solutions. The red circle symbol presented the hydrodynamic radius of original diluted SSZ-MG aqueous suspensions without pH adjusting.

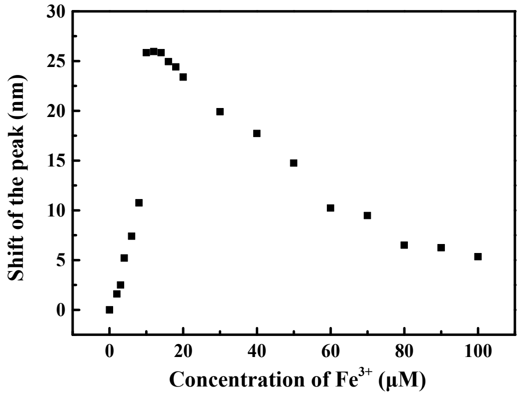

Figure S6. Wavelength shift of absorption peak at 362 nm of SSZ-MG aqueous suspensions as a function of Fe^{3+} concentration at 25 °C and pH of 5.6. The concentration of SSZ-MG aqueous suspensions was 0.174 mg/mL. The corresponding concentration of SSZ moieties ([SSZ]) in SSZ-MG aqueous suspensions was 23.09 μM.

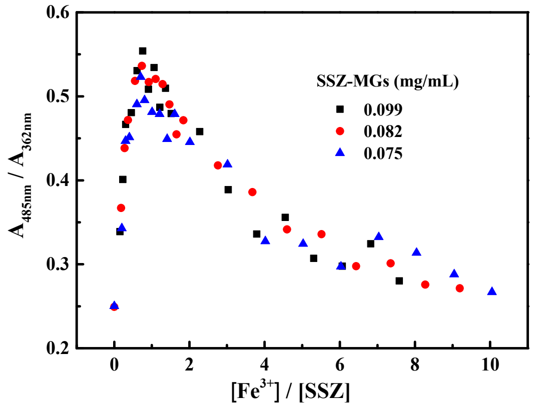

Figure S7. A_{485nm}/A_{362nm} ratios as a function of $[Fe^{3+}]/[SSZ]$ for SSZ-MG aqueous suspensions with various concentrations at 25 °C and pH of 5.6. The concentrations of SSZ-MG aqueous suspensions were 0.099, 0.082, and 0.075 mg/mL, respectively. The corresponding concentrations of SSZ moieties were 13.19, 10.88, and 9.95 μM, respectively.

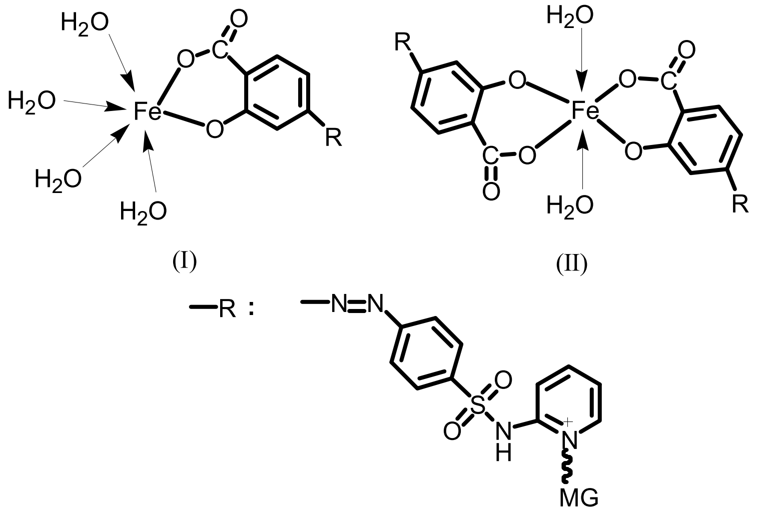

Figure S8. Possible coordination structures of SSZ moieties and Fe^{3+} ion.

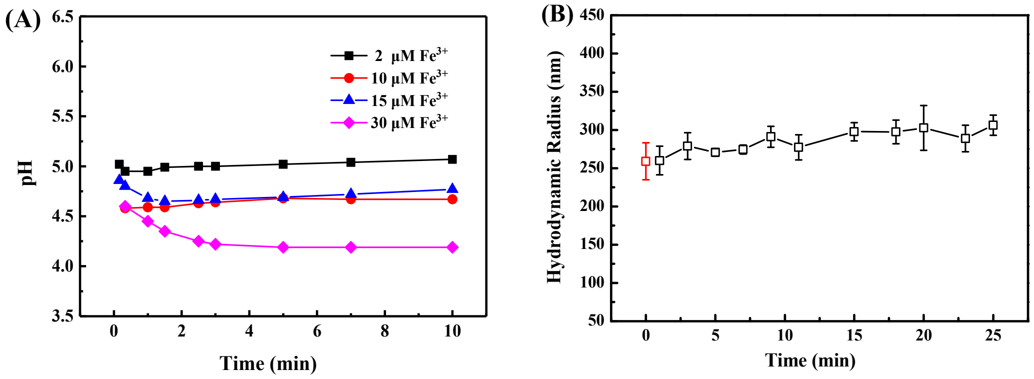

Figure S9. (A) The pH values of SSZ-MG aqueous suspensions (0.174 mg/mL) as a function of time monitored immediately after addition of 2, 10, 15, and 30 μM Fe^{3+} ions at 25 °C. **(B)** The hydrodynamic radius of SSZ-MG aqueous suspensions (0.174 mg/mL) as a function of time measured by DLS immediately after addition of 10 μM Fe^{3+} ions at 25 °C. The red symbol was the hydrodynamic radius of original SSZ-MG aqueous suspensions.

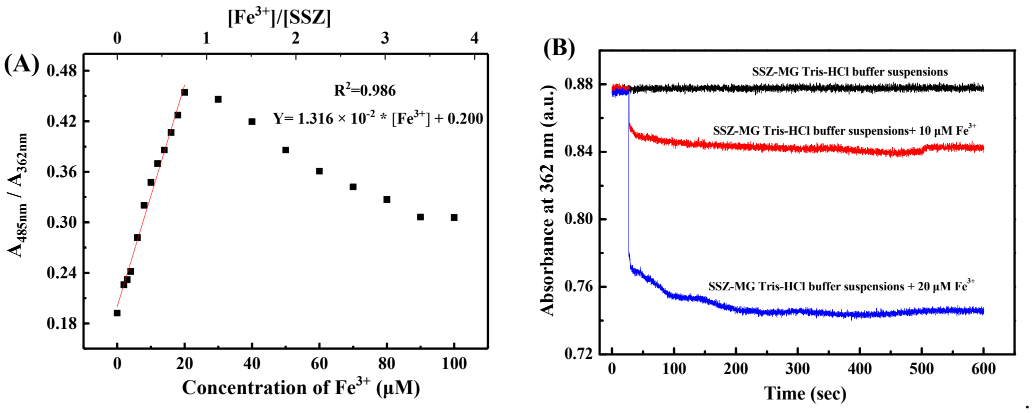

Figure S10. (**A**) The A_{485nm}/A_{362nm} ratio of SSZ-MG Tris-HCl buffer suspensions (0.196 mg/mL) as a function of Fe^{3+} concentration ([Fe^{3+}]) as well as [Fe^{3+}]/[SSZ] ratio. (**B**) Plot of absorption intensity at 362 nm of SSZ-MG Tris-HCl buffer suspensions (0.196 mg/mL) as a function of time after adding 10 and 20 μM Fe^{3+} ions.

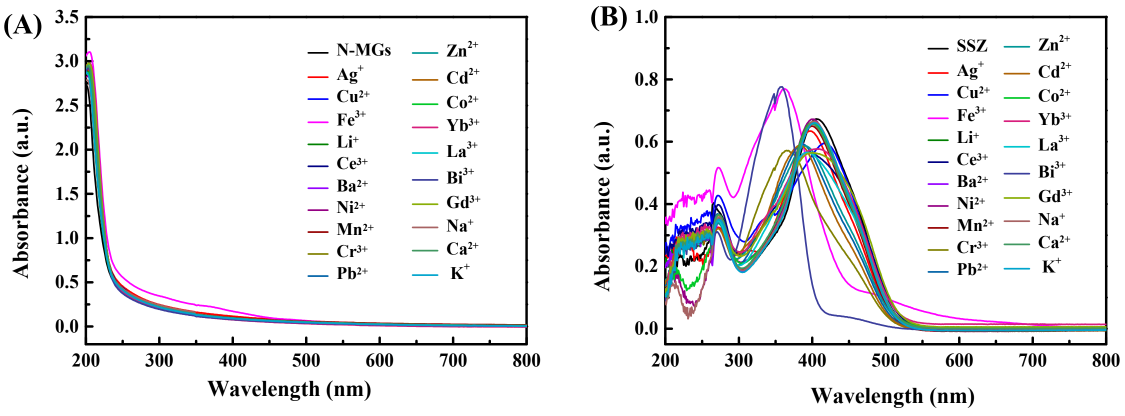

Figure S11. (**A**) The UV-vis absorption spectra of N-MG aqueous suspensions (0.099 mg/mL) after addition of 50 μM various metal ions, respectively, at 25 °C and pH of 5.6. (**B**) The UV-vis absorption spectra of SSZ molecules in DMF (0.012 mg/mL) with the presence of 50 μM various metal ions, respectively.

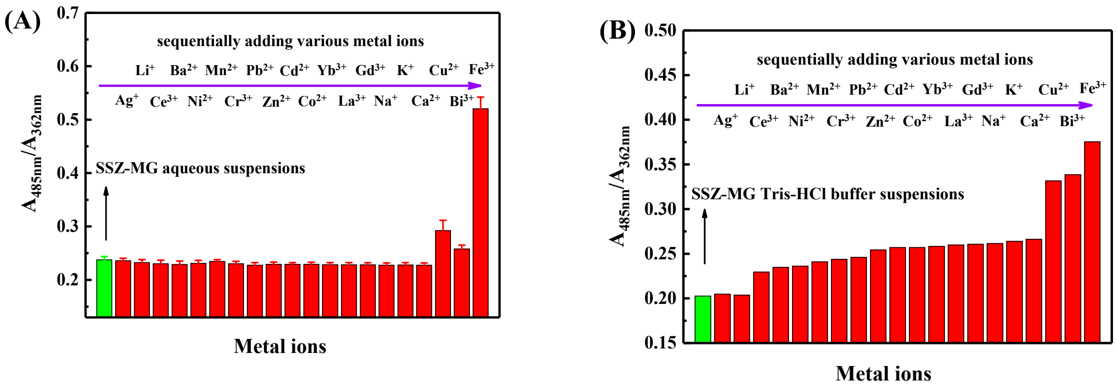

Figure S12. (**A**) The A_{485nm}/A_{362nm} ratios of SSZ-MG aqueous suspensions (0.196 mg/mL, pH 5.6) upon sequential addition of different metal ions (10 μM) at 25 °C. (**B**) The A_{485nm}/A_{362nm} ratios of SSZ-MG Tris-HCl buffer suspensions (0.196 mg/mL, pH 7.1) upon sequential addition of different metal ions (10 μM) at 25 °C. The addition sequences of metal ions were Ag^{+}, Li^{+}, Ce^{3+}, Ba^{3+}, Ni^{2+}, Mn^{2+}, Cr^{3+}, Pb^{2+}, Zn^{2+}, Cd^{2+}, Co^{2+}, Yb^{3+}, La^{3+}, Gd^{3+}, Na^{+}, K^{+}, Ca^{2+}, Cu^{2+}, Bi^{3+}, and Fe^{3+} for (A) and (B).

Table S1. Concentrations of Fe^{3+} ions spiked in deionized water, lake water, and drinking water from Yuquan campus of Zhejiang University as determined by UV-vis absorption spectroscopy with SSZ-MG aqueous suspensions (0.082 mg/mL) as the optical sensor.

Sample	[Fe^{3+}] Measured by Elemental Analysis (nM)	[Fe^{3+}] spiked (μM)	[Fe^{3+}] Measured by UV-Vis Spectroscopy (nM)
deionized water	0	4	3960 ± 76
		8	8079 ± 38
		10	10057 ± 59
lake water	23 ± 13	4	3938 ± 90
		8	8111 ± 41
		10	9994 ± 148
drinking water	43 ± 10	4	4100 ± 64
		8	8037 ± 133
		10	10088 ± 45
